# Evaluation of a digital triage platform in Uganda: A quality improvement initiative to reduce the time to antibiotic administration

**DOI:** 10.1371/journal.pone.0240092

**Published:** 2020-10-02

**Authors:** Victor Lee, Dustin Dunsmuir, Stephen Businge, Robert Tumusiime, James Karugaba, Matthew O. Wiens, Matthias Görges, Niranjan Kissoon, Sam Orach, Ronald Kasyaba, J. Mark Ansermino

**Affiliations:** 1 Department of Anesthesiology, Pharmacology and Therapeutics, University of British Columbia, Vancouver, British Columbia, Canada; 2 Center for International Child Health, BC Children’s Hospital Research Institute, Vancouver, British Columbia, Canada; 3 Holy Innocents Children's Hospital, Mbarara, Uganda; 4 School of Population and Public Health, University of British Columbia, Vancouver, British Columbia, Canada; 5 Department of Pediatrics, University of British Columbia, Vancouver, British Columbia, Canada; 6 Uganda Catholic Medical Bureau, Kampala, Uganda; Texas A&M University, UNITED STATES

## Abstract

**Background:**

Sepsis is the leading cause of death in children under five in low- and middle-income countries. The rapid identification of the sickest children and timely antibiotic administration may improve outcomes. We developed and implemented a digital triage platform to rapidly identify critically ill children to facilitate timely intravenous antibiotic administration.

**Objective:**

This quality improvement initiative sought to reduce the time to antibiotic administration at a dedicated children’s hospital outpatient department in Mbarara, Uganda.

**Intervention and study design:**

The digital platform consisted of a mobile application that collects clinical signs, symptoms, and vital signs to prioritize children through a combination of emergency triggers and predictive risk algorithms. A computer-based dashboard enabled the prioritization of children by displaying an overview of all children and their triage categories. We evaluated the impact of the digital triage platform over an 11-week pre-implementation phase and an 11-week post-implementation phase. The time from the end of triage to antibiotic administration was compared to evaluate the quality improvement initiative.

**Results:**

There was a difference of -11 minutes (95% CI, -16.0 to -6.0; p < 0.001; Mann-Whitney *U* test) in time to antibiotics, from 51 minutes (IQR, 27.0–94.0) pre-implementation to 44 minutes (IQR, 19.0–74.0) post-implementation. Children prioritized as emergency received the greatest time benefit (-34 minutes; 95% CI, -9.0 to -58.0; p < 0.001; Mann-Whitney *U* test). The proportion of children who waited more than an hour until antibiotics decreased by 21.4% (p = 0.007).

**Conclusion:**

A data-driven patient prioritization and continuous feedback for healthcare workers enabled by a digital triage platform led to expedited antibiotic therapy for critically ill children with sepsis. This platform may have a more significant impact in facilities without existing triage processes and prioritization of treatments, as is commonly encountered in low resource settings.

## Introduction

The global burden of mortality in children remains high, with 5.3 million deaths in children under five years of age in 2018 [1]. Most of these deaths were from infectious diseases such as pneumonia, diarrheal diseases, and malaria [2, 3]. There is a higher prevalence of infectious diseases and a higher sepsis case-fatality rate in low and middle-income countries (LMICs) [4], hence improving outcomes from sepsis among children in low-resource settings is crucial to achieving the third Sustainable Development Goal of reducing under-five mortality [5].

Current evidence-based guidelines for sepsis care recommend patients receive intravenous antibiotics (IVA) within one hour of presentation [6]. Delay in IVA administration is associated with increased in-hospital and post-discharge mortality for adults [7, 8], while frugal bundles of care that include IVA within one hour have significantly reduced mortality for children [9]. While these recommendations were based on evidence from highly resourced environments with intensive care support, early administration would be more important in poorly resourced, non-resilient settings without the ability to support children who develop multisystem failure [10]. Triage is important for prioritizing these sickest children.

Although the World Health Organization’s Integrated Management of Childhood Illness manual (IMCI) and Emergency Triage Assessment and Treatment protocol (ETAT) aid in diagnosis and triage [11, 12], successful implementation is stymied by significant hospital resource limitations including the dedicated time required for healthcare workers to undergo training [13, 14]. Electronic technology using mobile devices may circumvent some barriers and may improve the adherence to IMCI protocols [15, 16]. However, an electronic system designed for patient triage has not previously been implemented in LMICs. With this in mind, we developed a digital triage platform for frontline healthcare providers.

Our platform included a mobile application with danger signs and predictive risk scoring algorithms [17, 18], which were driven by clinical signs and symptoms, collected through multiple choice questions, and vital signs, measured from a mobile pulse oximeter attachment and a software-based respiratory rate counter [19, 20]. The platform also included a dashboard which provided real-time patient information for optimized resource allocation and patient prioritization. The goal of the platform was to rapidly identify critically ill children with sepsis or a heightened risk of developing sepsis, so that resources could quickly and appropriately be allocated to them. We conducted an interrupted time series study, as part of a quality improvement initiative, with the primary research question of whether a digital triage platform can reduce the time to IVA administration for children seen in an LMIC outpatient department.

## Methods

This was a quality improvement initiative, occurring over an 11-week pre-implementation period for baseline data collection (July to October 2018), a 5½-week implementation and training phase, and an 11-week post-implementation evaluation period (November 2018 to January 2019).

Ethics approval for this project was obtained from Mbarara University of Science and Technology Institutional Review Committee (reference no. 11/02-18). As a quality improvement initiative, ethics approval was exempted by the University of British Columbia and individual consent was waived by the Mbarara University of Science and Technology.

### Setting and population

We conducted this study at the Holy Innocents Children's Hospital (HICH) in Mbarara, Uganda. HICH is a private, non-profit, faith-based hospital offering subsidized fee-for-service inpatient and outpatient care for children up to sixteen years of age. The outpatient department provides care for over 25,000 children yearly, and employs two pediatricians, four general physicians, three clinical officers, and thirty-four nurses on rotation. It is open every day for 24 hours, with nurse shifts running for 12 hours and shift changes at 8:00 and 20:00. There are three consultation rooms and one treatment room.

The pre-implementation triage procedure at HICH was based on the case by case clinical judgement of a nurse. Triage procedures were inconsistently applied, but in general, children with severe vomiting, severe diarrhea, or high temperature were triaged as priority, while emergency triage was reserved for more severe cases such as unconsciousness, convulsing, obvious respiratory distress, or severe trauma. Neonates were triaged as an emergency if there was suspicion of serious infection, sepsis, or premature birth. Patients triaged as emergency were typically transferred directly to the treatment room. Patients identified with a high temperature were given an oral or rectal antipyretic in the triage room. Other patients, including priority patients, waited in order of arrival and were generally seen on a first-come, first-served basis.

All patients presenting at the HICH’s outpatient department were included in the study, except for children presenting for vaccinations, elective surgery, or change of wound dressings.

### Triage platform

**Fig 1:**
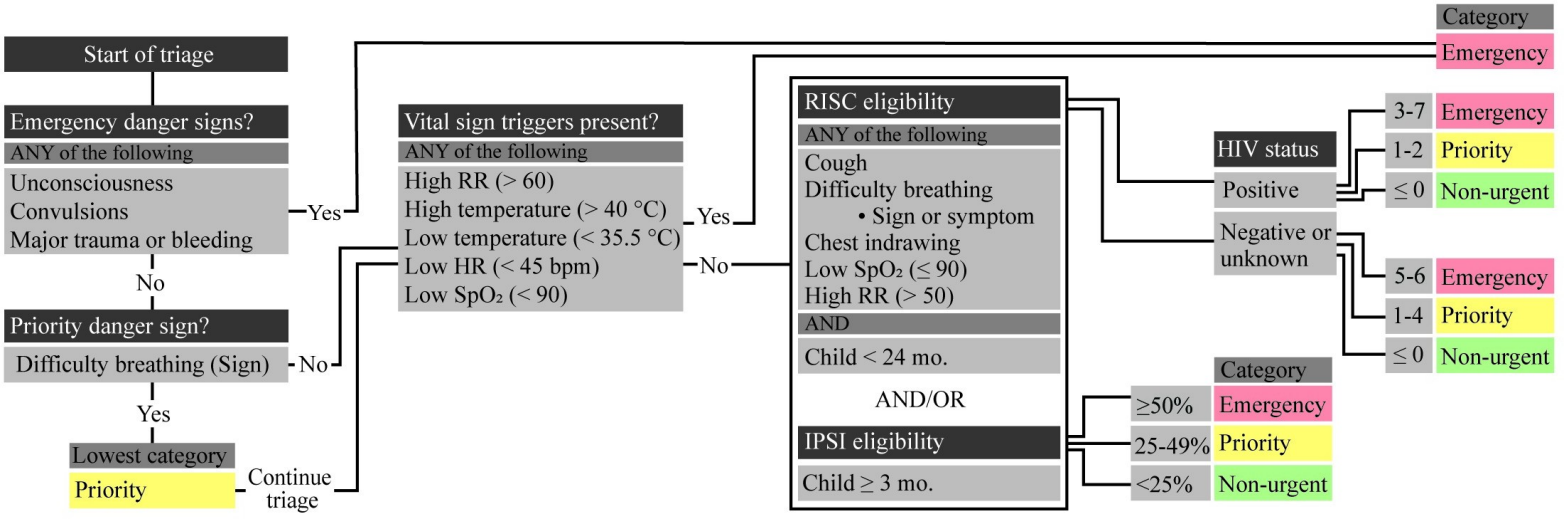
Triage algorithm for patient classification upon arrival. RR, respiratory rate; HR, heart rate; SpO_2_, oxygen saturation; IPSI, Interrupting Pathways to Maternal, Newborn, and Early Childhood Sepsis Initiative model [17]; RISC, Respiratory Index of Severity in Children [18].

We developed a digital triage platform that included a mobile application running on Nexus 6 Android phones (Motorola Mobility, Chicago, USA), combined with a pulse oximetry sensor attachment (LionsGate Technologies, Vancouver, Canada), to collect clinical symptoms (N = 11), signs (N = 5), and vital signs (N = 4). The triage algorithm identified danger signs, followed by a cascade of risk scores and single variable threshold values to triage each child as non-urgent, priority, or emergency (Fig 1).

**Fig 2:**
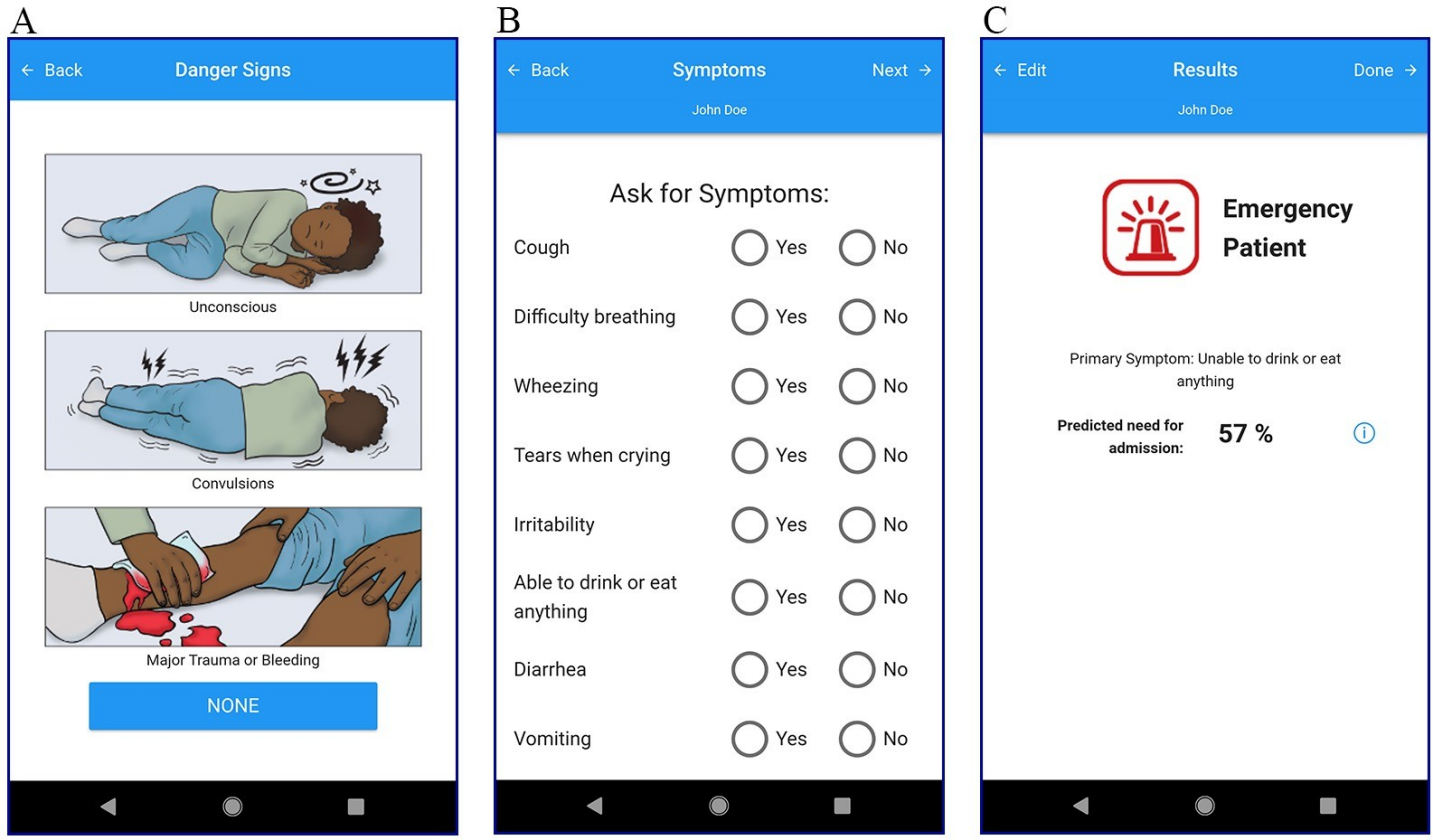
Screenshots of the mobile triage application. (A) shows the danger signs, (B) shows the list of symptoms, and (C) shows the final triage category.

Danger signs, which were chosen in collaboration with local clinical experts, were shown first. If no danger signs were present or the nurse decided to continue using the app after a notification displayed the patient’s emergency categorization based on the danger signs, the triage process continued for clinical signs and symptoms to be entered through a series of multiple-choice questions (Fig 2). Oxygen saturation (SpO_2_) and heart rate (HR) were measured on the smartphone through an integrated app, PhoneOx [19], with a pulse oximeter attachment (Envisen Industry, Shenzhen, China) connected via a DB9 to USB-B adaptor (LionsGate Technologies, British Columbia, Canada). Respiratory rate (RR) was measured by another integrated app, RRate [20], through tapping of the touch screen.

The Interrupting Pathways to Maternal, Newborn, and Early Childhood Sepsis Initiative (IPSI) model [17], and the Respiratory Index of Severity in Children (RISC) score [18], were used to categorize the risk of children according to the severity of their illnesses. The IPSI model was applied to all children 3 months or older and predicted the need for admission based on age, temperature, RR, SpO_2_, the presence of a cough, difficulty breathing, chest indrawing, lethargy, irritability, and diarrhea. The RISC score was applied to children under two years of age with indications of lower respiratory chest infections and predicted mortality based on age, weight, SpO_2_, the presence of chest indrawing, wheezing, the inability to drink or eat, and HIV status. Thresholds for the individual risk scores (detailed in Fig 1) were conservatively chosen.

**Fig 3:**
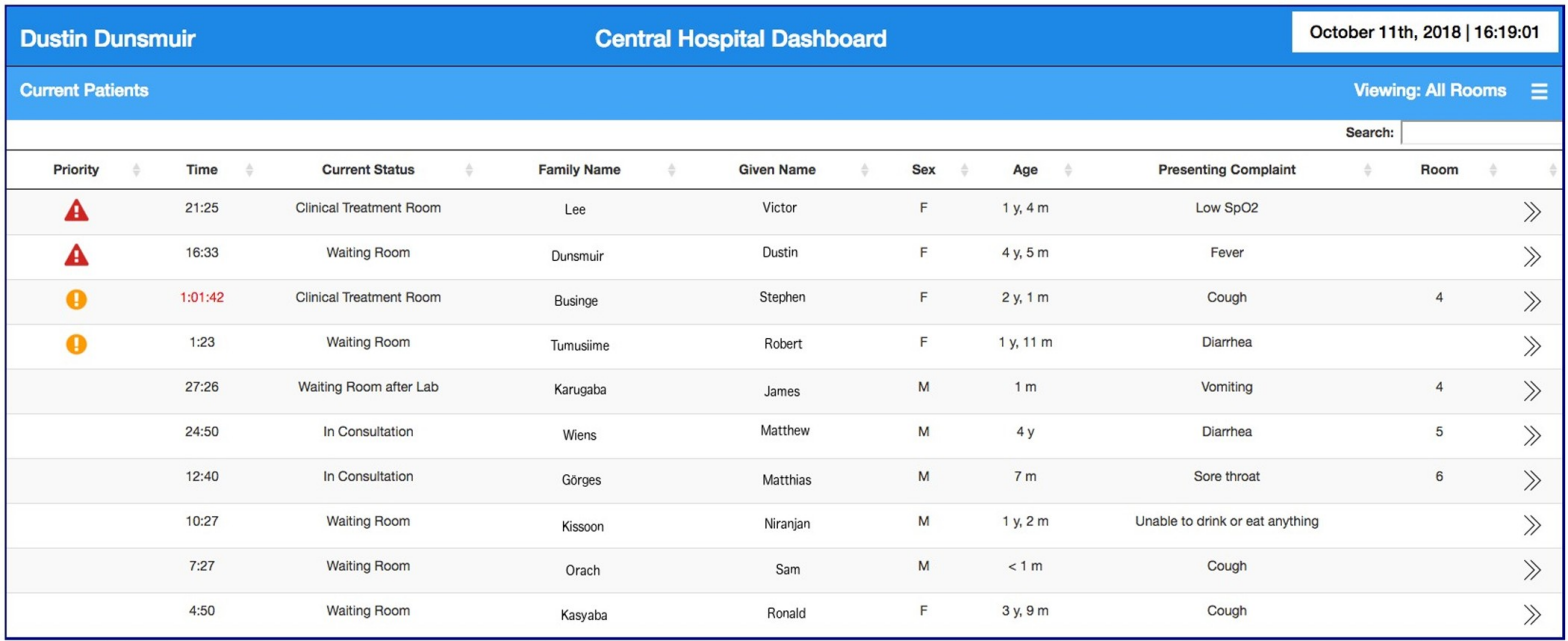
Clinician dashboard. Patient prioritizations as warning symbols in the first column, time elapsed since patient arrival in the second column, current patient location in the third column, and other summary information in the remaining columns were shown. Full triage details were available on a separate page by clicking the patient’s row.

After triage, patient details, which included the patient’s age, presenting complaint, triage time, and triage category, were sent to an accompanying dashboard that allowed clinicians to appropriately prioritize patients (Fig 3). The web-based dashboard was accessible on the local network through desktop computers and Android tablets located in the consultation and treatment rooms for healthcare workers to view and record patient location (e.g. waiting room, consultation room) and treatments (e.g. intravenous fluids, antibiotics). A warning appeared if the elapsed time for priority or emergency triage patients exceeded one hour.

### Implementation and usability

The implementation and transition phase (lasting 5½ weeks) included the adjustment of the triaging tool and dashboard to meet the needs of the nurses and clinicians at HICH. This was a collaborative and interactive process to increase usability and local support for the system. The triage platform was customized to communicate with the HICH’s existing electronic health record (EHR), an open-source hospital information system called care2x (care2x.org), so that all existing measurements were included to prevent redundant data entry. The patient listing on the dashboard was adjusted to include only the information deemed most important for identifying and prioritizing a patient.

During the implementation and transition phase, a usability evaluation was conducted. A simulated child was entered into the mobile application, while nurses and clinicians located information on the dashboard and updated the status of the child. We gathered feedback through direct observation of the users as well as providing a forum for follow-up discussions, after which several improvements were made to the platform. Prior to the start of the post-implementation period, we conducted follow-up interviews with triage nurses and clinicians in order to assess any usability issues or desired improvements that were apparent once the platform had been implemented in the facility.

During the post-implementation period, we provided weekly feedback reports in the form of key summaries, including the duration of triage, treatment times, and attributes of children admitted to the facility. The dashboard also included live updating bar charts and line graphs with these same details for continuous quality improvement.

### Data collection

In the pre-implementation period, patients were registered in the facility's EHR system, where registration information including the patient’s name, age, and district was recorded. Triage nurses measured and recorded anthropometric data, and all treatment times were recorded manually by nurses in the treatment room.

In the post-implementation period, patients were registered in the facility's EHR system then triaged by a nurse using the mobile application, which automatically acquired the patient’s name, age, and district from the EHR. If the triage nurse decided that a patient clearly needed immediate care (e.g. unconsciousness, severe trauma), the mobile application was not used, and the patient was brought directly to the treatment room. Treatment data of patients who were not triaged by the mobile application were not collected.

All electronic study data were temporarily stored on HICH’s servers, and de-identified data were sent securely with rsync (available from https://rsync.samba.org/) to servers at the University of British Columbia daily for further analysis.

### Evaluation and analysis

The differences in patient characteristics between the two periods were compared using Mann-Whitney U tests (age, weight, and height) and chi-squared tests (sex and antibiotic usage). Nurses had the option to skip entering these patient characteristics, so missing data were excluded from numerical summaries. All continuous variables were expressed as medians with interquartile ranges (IQR), and a two-tailed alpha level of less than 0.05 was considered significant for all statistical tests. Data pre-processing and analysis were completed in R 3.6.1 [21].

The primary outcome measure was the time from the end of triage to administration of IVA. In addition, we compared the time to first treatment for the particularly vulnerable patients who received both intravenous fluids and antibiotics. The proportion of patients who received IVA and the time to IVA within each triage category were compared. All times were compared with Mann-Whitney U tests, and proportions were compared with Fischer’s exact tests. The number of patients who received IVA after waiting over one hour were compared using a chi-squared test.

Segmented regression analysis of the interrupted time series of the pre- and post-implementation periods was used to assess immediate effects and effects over time, controlling for prior trends that may have continued without our intervention [22]. We also compared the estimated times at end of the post-implementation period with the expected counterfactual value, had the pre-implementation trend continued as if the intervention never occurred [22]. Quantile regression was used to estimate the median differences.

To assess whether the dashboard affected the prioritization of children, the compliance towards the triage platform’s prioritization was measured. This was estimated by comparing the triage order of all children and the order in which children were seen. The triage prioritization was considered adhered to if the patient was the next to be seen, with five minutes allowing for patient movement and for the dashboard to be updated. Patients who did not wait to be seen regardless of their triage prioritization, as they were the sole child waiting at the given time, were not considered in this calculation.

## Results

There were 5,698 children registered at the hospital during the pre-implementation phase and 5,522 registrations during the post-implementation phase, of which 413 were not triaged by the mobile application. Patient characteristics of the two cohorts were not significantly different, other than fewer children receiving IVA in the post-implementation period (Table 1).

**Table 1:** Patient characteristics.

Variable	Pre-implementation	Post-implementation	P-Value
Triaged, n	5,698	5,104	
Sex male, n (%)	3,144 (55)	2,756 (54)	0.226
Age, median mos. (IQR)^a^	23.8(9.4–53.0)	24.0(10.2–52.4)	0.459
Height, median cm (IQR)^b^	88.0(73.0–109.0)	87.0(73.0–108.0)	0.234
Weight, median kg (IQR)^c^	12.0(9.0–17.0)	11.5(8.7–17.0)	0.183
Geographical district, n (%)			
Mbarara	3377 (59)	2989 (59)	
Isingiro	678 (12)	598 (12)	
Kiruhura	470 (8)	416 (8)	
Ntungamo	272 (5)	229 (4)	
Bushenyi	199 (3)	198 (4)	
Missing	71 (1)	124 (2)	
Other	631 (11)	674 (11)	
Intravenous antibiotics, n (%)	538 (9.4)	351 (6.9)	< 0.001

^a^There were 14 negative and 17 missing ages;

^b^29 heights under 25 cm, 5 over 200 cm, and 757 missing heights; and

^c^11 weights under 1 kg, 1 over 150 kg, and 487 missing weights that were excluded.

Of the 538 children who received IVA during the pre-implementation phase, 41 (7.6%) IVA times were excluded from analysis: One child had no triage time recorded. Twelve children had a time to IVA over five hours because treatment was intentionally delayed. Twenty-eight children were excluded because they had IVA times before recorded triage times. Therefore, there were 497 times analyzed in the pre-implementation phase.

The median time to IVA was 51 minutes (IQR, 27.0–94.0) pre-implementation and 44 minutes (IQR, 19.0–74.0) post-implementation, representing a difference of -11 minutes (95% CI, -16.0 to -6.0; p < 0.001; Table 2). Individual triage groups all had a decrease in time to IVA, except for the priority group, which had a 14 minute increase in time to IVA (Table 2). Of the children triaged in the post-implementation phase, the triage platform’s prioritization was followed in 78% of emergency cases and 40% of priority cases. In the post-implementation phase, there was a median wait time of 7 minutes (IQR, 3.0–15.0) from registration to triage, and the triage process took a median of 4 minutes (IQR, 2.0–5.0).

**Table 2:** Triage categorization and intravenous antibiotic outcomes.

Variable	Pre-implementation	Post-implementation	Effect Size^a^	P-Value
Triaged, n	5,698	5,104		
Received IVA, n (%)	538 (9.4)	351 (6.9)	OR: 0.71 (0.61, 0.82)	< 0.001
Time to IVA, median min. (IQR)	51.0 (27.0–94.0)	44.0 (19.0–74.0)	-11.0 (-16.0, -6.0)	< 0.001
Emergency, n	32	215	OR: 7.78 (5.34,11.70)	< 0.001
Received IVA, n (%)	13 (40.6)	88 (40.9)	OR: 0.99 (0.42, 2.23)	1.0
Time to IVA, median min.(IQR)	50.0 (14.0–83.0)	9.5 (2.0–26.8)	-34.0 (-58.0, -9.0)	< 0.001
Priority, n	426	402	OR: 1.11 (0.96, 1.28)	0.147
Received IVA, n (%)	160 (37.6)	50 (12.4)	OR: 0.24 (0.16, 0.34)	< 0.001
Time to IVA, median min. (IQR)	26.5 (17.8–40.8)	47.5 (24.3–84.5)	14.0 (5.0, 25.0)	0.002
Non-urgent, n	5,240	4,487	OR: 0.64 (0.56, 0.72)	< 0.001
Received IVA, n (%)	324 (6.2)	213 (4.7)	OR: 0.76 (0.63 0.91)	0.002
Time to IVA, median min. (IQR)	71.0 (42.8–107.0)	57.0 (34.0–86.0)	-12.0 (-20.0, -5.0)	< 0.001

^a^Differences compared with Mann-Whitney *U* test presented for antibiotic times. IVA, Intravenous antibiotics.

Children who received both intravenous fluids and antibiotics had a difference of -18 minutes (95% CI, -34.0 to -4.0; p = 0.017; Mann-Whitney *U* test) in time to first treatment, decreasing from 74 minutes (IQR, 38.0–98.0; n = 59) pre-implementation to 51 minutes (IQR, 25.5–80.3; n = 50) post-implementation.

**Fig 4:**
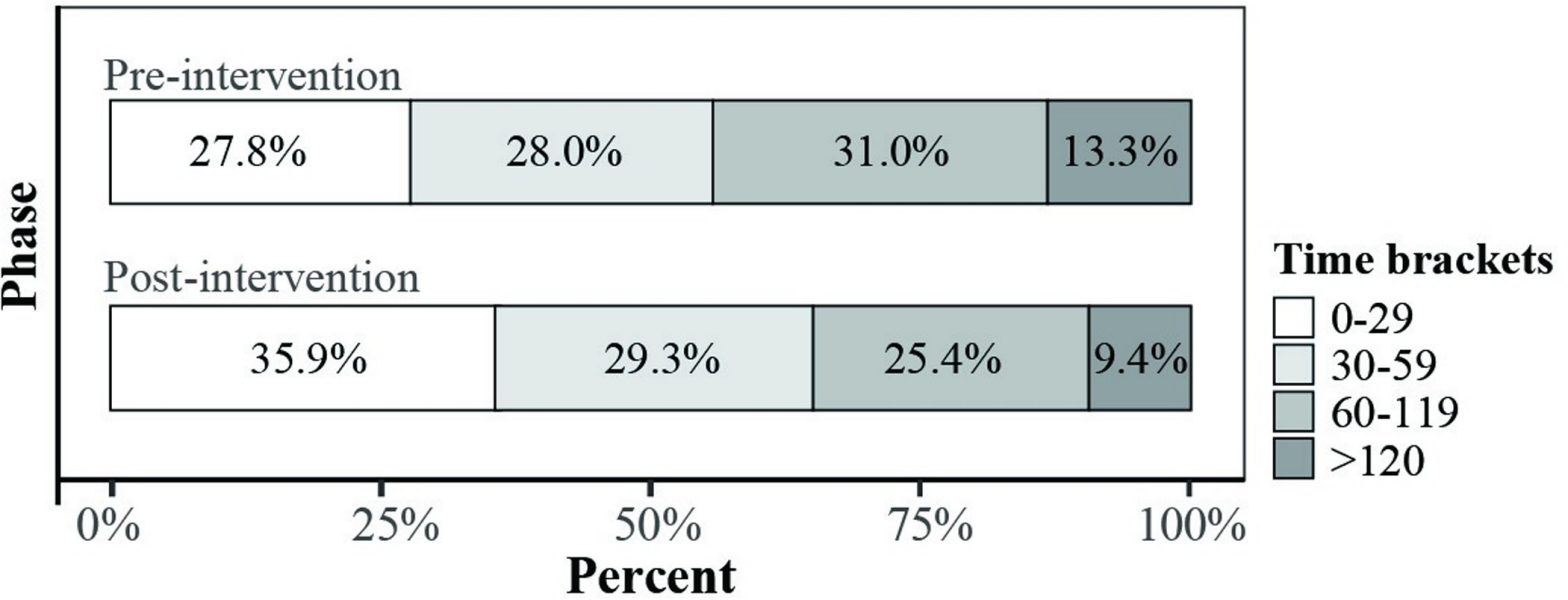
Percentage of children who received intravenous antibiotics for each time bracket, pre- and post-implementation.

During the pre-implementation phase, 44.3% of children waited one hour or longer for IVA (Fig 4). This decreased by 21.4% (p = 0.007) to 34.8% in post-implementation phase. There was also an associated 22.3% (p = 0.015) increase in patients who received IVA within 30 minutes, from 27.8% to 35.9%.

**Fig 5:**
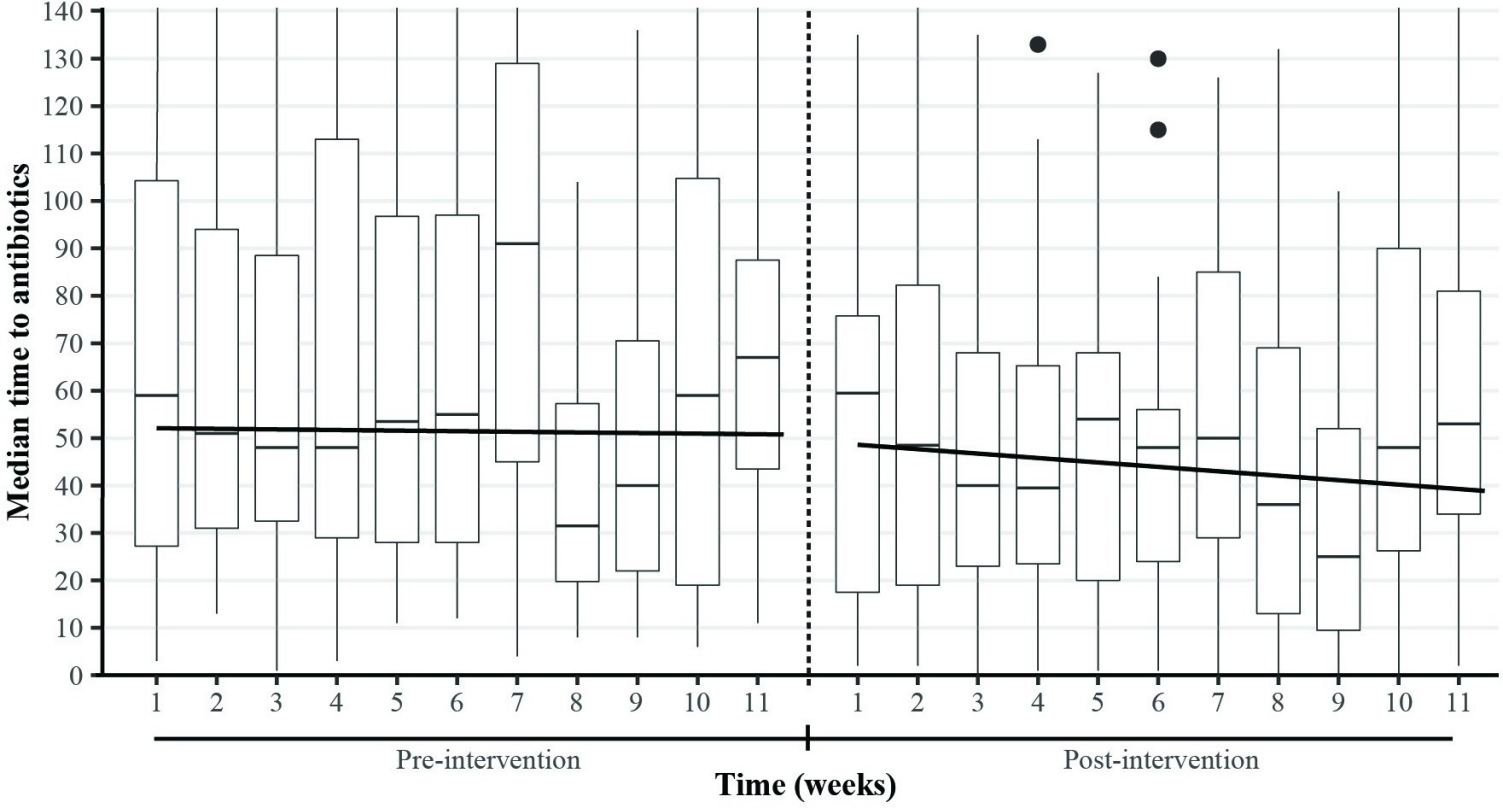
Segmented regression of the time to intravenous antibiotics for all children, overlaid with boxplots of weekly antibiotic wait times.

Segmented regression analysis demonstrated an immediate 2-minute (p = 0.834) decrease between the end of pre-implementation and the start of post-implementation phases (Fig 5). The slope changed 48 seconds per week (p = 0.589), from -8 seconds per week pre-implementation to -56 seconds per week post-implementation. If the initiative had not occurred, but the pre-implementation trend continued, the estimated median time at the end of the evaluation period would be 49 minutes. The true median time at the end of the evaluation period from the linear regression was 39 minutes.

## Discussion

This study evaluated a digital triage platform implemented as a quality improvement initiative targeting the expedited treatment of critically ill children with sepsis in a low-resource setting. The platform reduced the time to antibiotic administration, especially in the highest risk children, and increased the proportion of children who received antibiotics under one hour, demonstrating the potential for data-driven electronic triage and technology in quality improvement.

### Clinical interpretation

The timely administration of antibiotics has mortality benefits, especially for the sickest children who have the most to gain. Several studies in adults have shown each hour of delay in administration of IVA was associated with increased mortality [7–9, 23–25]. Specifically, Liu *et al*. [25] found that all adult sepsis patients have increased odds of mortality with IVA delay and patients with septic shock receive a 1.8% mortality increase per hour of IVA delay. The largest study of pediatric sepsis with 1179 children in New York hospitals also showed that a frugal bundle of care which included antibiotics within one hour resulted in a significant decrease in mortality [9]. Furthermore, rapid treatment may be particularly important for patients who present at a late stage, as is commonly encountered in low-resource settings where health-seeking behaviour is poor, and in rural areas where the use of hospital care is strongly associated with travel times [26–29].

Even though overall reduction in time to IVA was modest, the critical patients prioritized as emergency benefitted from a 34-minute reduction in the time to IVA after implementation of the digital triage platform, receiving IVA in a median time of 9.5 minutes. The severely ill children who may not have previously been identified by triage nurses, may be prioritized by the triage application as emergency patients, and have benefitted from expedited treatment. This group of children may be reflected by the significant decrease in the proportion of children who waited more than one hour and increase in the proportion of children who waited less than half an hour until antibiotic administration. There was a significant increase in time for the children categorized as priority. The new triage processes caused a redistribution of patients within the three triage categories, with far more children prioritized as emergency post-implementation. There was also a decrease in the likelihood that priority patients would receive IVA from pre-implementation (37.6%) to post-implementation (12.4%). The increase in time to IVA for those 12.4% of priority patients that still received IVA is not surprising, given the significant increase in the emergency patients who were seen first.

### Segmented regression

We used segmented regression, which improves the internal validity of longitudinal quasi-experimental studies [22, 30], to analyze the time series data. These analyzes found that the level decreased immediately after implementation and the slope became magnitudes more negative (Fig 5), suggesting that prior trends were not confounding the results. The lack of statistical significance is due to the segmented regression being completed on all data points rather than monthly means. While the latter method is typically used in this type of analysis [22, 30], we decided that modeling every time point would be more illustrative than modeling monthly averages of the 2½-month post-implementation period.

### Pre-implementation performance at Holy Innocents Children’s Hospital

The potential impact of the initiative was limited by already highly efficient performance in the pre-implementation phase. In comparison, a recent sepsis care improvement initiative in a North American tertiary care children’s hospital decreased the mean time for antibiotic administration from 154 to 114 minutes [31]. In a further study at a resource rich hospital, only 61% of children with severe sepsis or septic shock received IVA in under three hours [32]. As such, the digital triage platform is likely to have a more significant impact in low-resource facilities with less efficient treatment procedures, where chronic overcrowding due to inadequate facilities, personnel, and supplies is common [6].

### Implications for mHealth and triage

Our results show that a mobile application can contribute to improved child healthcare in emergency department management in a low-resource setting. Computer-based decision support systems have long been used to improve clinical care [33]. Gatewood *et al*. [34] showed that electronic alerts screening for sepsis at triage can expedite the delivery of care in a high-resource setting. There is increasing evidence that technology can improve healthcare systems and healthcare delivery, but there are few mHealth projects that facilitate care or operate at scale in LMICs [35]. Of the few projects in low-resource countries, studies have shown the feasibility and effectiveness of mobile devices with electronic IMCI protocols [15, 16]. In particular, Ginsburg *et al*. [16] developed and conducted a usability field test with an app designed to improve the diagnosis and management of pneumonia with established guidelines. In this study we demonstrated the potential for clinical prediction models, implemented within a mobile application, for triage. As entry level smartphones are readily available, or at least affordable compared to other medical devices, a mobile application-based triage platform can be widely implemented in low-resource settings. The cost associated with implementation in a new facility is limited to smartphones, basic computers (as only a web browser is needed) or tablets, and a local network.

Additionally, a digital triage platform overcomes many of the challenges of traditional triage protocols. The ETAT protocols have high barriers of initiation because of implementation challenges, which include the intensive training and commitment required of the healthcare providers, together with facility challenges, including the use of conflicting guidelines by different healthcare workers [13]. A triage app hiding a cascade of risk scoring algorithms and the inclusion of an automated respiratory counter reduces the need for memorization and simplifies the entire triage process.

### Clinical outcomes of quality improvement efforts

Targeting a process improvement, such as time to IVA administration, can be used to drive a process of quality improvement that extends beyond triage and IVA therapy alone. The goal of any quality improvement initiative should be to improve clinical outcomes, such as a reduction in mortality; however, the study was not sufficiently powered to demonstrate this, and outcomes were not recorded. Improving sepsis care requires the adoption of constantly evolving evidence-based practices, evaluated through monitoring both individual process and outcomes measures [36]. Continuous performance measurement and feedback is a key element of many successful quality improvement efforts [37]. A strength of the mobile application and clinical dashboard is that it serves as a platform for continuous cycles of quality improvement though its data collection and feedback mechanisms, contributing to the culture of providing high quality care.

### Limitations

A significant barrier to the widespread implementation of the digital platform is the availability of smartphones and the requirement for computer infrastructure. The digital platform was easily implemented at HICH because the facility already used computers, a wireless network, and an EHR. Although technology is rapidly becoming more accessible, modifications to the platform may be necessary for use in healthcare facilities with less pre-existing technological resources. Additionally, a review in 2013 showed that less than one-third of healthcare facilities in sub-Saharan Africa have a reliable electricity source [38], but this systemic issue is improving with time.

The digital platform’s prioritization was not always followed. One factor that impacted this calculation was the administration of antipyretics for all patients identified with a high temperature while still in the triage room. Although the doctors were aware of antipyretics being given, interventions in the triage room were not recorded on the digital platform, so the patient’s prioritization may have been altered after this consideration. The imperfect adherence to the prioritization may also be the intentional actions of triage nurses, which may reflect the shortcomings of the digital platform’s predictions. Some conditions recognized by triage nurses may not be included or be considered by the predictive algorithm. Another possibility may simply be poor compliance with following the triage categorization, demonstrating the difficulty of changing the culture of receiving care in the order of arrival. We did not implement any specific processes to ensure that the triage categories were followed; instead healthcare providers were permitted to use the platform how they found appropriate. Future work will involve designing and implementing processes to improve the adherence to the platform’s triage categorization, such as the automated tracking of patient movement and treatment times. Additionally, a dashboard displaying the triage prioritizations to patients in the waiting area may help facilitate the adherence to triage prioritizations. We will test these additional features with an updated triage algorithm at public tertiary facilities in Uganda and Kenya.

The digital platform was evaluated through the process measure of time to IVA administration, but the appropriateness of IVA administration was not considered. The triage algorithms predicted children with the highest risk of admission and developing sepsis, but not all will necessarily require IVA. Since the IVA usage was based on a physician’s clinical decision, the triage platform may have had an impact on physician prescribing practices, contributing to the apparent reduction in the number and proportion of children receiving IVA. Whether the reduction is due to failures in recording all antibiotic doses given, prescribing antibiotics more appropriately, denying antibiotics when necessary, or less children requiring antibiotics is unknown. We excluded 28 children from analysis in the pre-implementation phase who had IVA times before recorded triage times, since children who required immediate care were brought directly to the treatment room without routine triage, and their triage data entered after. Nurses continued to bring patients who clearly required immediate care directly to the treatment room throughout the post-implementation phase, but treatment information cannot be entered on our dashboard if a patient was not initially triaged. There were 413 children who were not triaged post-implementation with the application; we suspect this group includes a higher proportion of high acuity patients because the lengthened triage process may be a deterrent for the application’s use, but no data was collected on the group. Further investigation on this is required, and the platform would be strengthened by the addition of electronic treatment recommendations.

The time to IVA administration may be inaccurate due to the use of manual documentation of treatments on the dashboard by nurses. The documented IVA time may be affected by whether times were recorded before, during or after therapy, as well as inadequate staffing, which may have caused delayed recordings of treatment times during busier periods. There may also be inaccuracies with the recorded time of first contact. Only the calculated measure of compliance towards triage prioritizations were based on that time, but if there were inaccuracies in recording, the proportions of triage prioritizations followed may have been underestimated. In future research, we will use a low-cost automated tracking system to robustly capture patient first contact, location, and treatments. This will also eliminate the need for healthcare workers to manually update the dashboard, streamlining the implementation and use of the platform.

Finally, inherent weaknesses of an interrupted time series study include the effects of seasonality on disease and whether improvements are sustained over time. While the wet season (Oct-Nov) in Mbarara coincided with both periods, a comparison between the pre- and post-intervention periods remains limited by the effects of seasonality on the incidence of disease. The conditions treated in the treatment room or medical ward were not collected, and the seasonality of different pathogens would influence the specific process measure of time to IVA. We acknowledge this limitation but believe that targeting IVA in a quality improvement initiative was the best option for its correlation with mortality benefits. The digital platform use continued after the official evaluation period so a follow-up analysis can assess whether the improvement in process measures is sustained.

## Conclusion

We demonstrated that a digital triage platform aimed at improving care, through patient prioritization and continuous data-driven feedback, can successfully reduce the time to antibiotic administration in a hospital outpatient department in Uganda. This digital triage platform has the potential to reduce child mortality in low resource settings, although further work is required to evaluate its impact in hospitals with lower levels of technology infrastructure and less well-defined triage and treatment processes.

## Supporting information

S1 Data Triage and treatment time data. (CSV)
